# Assessing the Impact of Boer Goat x Indigenous Goat Crossbreeding on Reproductive Performance and Farmer Perceptions in Southern Ethiopia

**DOI:** 10.1155/2024/6637667

**Published:** 2024-07-17

**Authors:** Kebede Habtegiorgis, Debir Legesse, Mohamed Bikamo

**Affiliations:** ^1^ Areka Agricultural Research Center, P.O. Box 79, Areka, Ethiopia; ^2^ Hawasa Agricultural Research Center, P.O. Box 6, Awassa, Ethiopia

## Abstract

Crossbreeding aims to increase the productivity of local or indigenous animals by introducing exotic breeds. This study aims to assess the effects of crossbreeding using 50% Boer bucks crossed with Ethiopian indigenous Woyto-Guji goats and to evaluate farmers' perceptions towards crossbred kids. Data were collected from five purposively selected districts in southern Ethiopia. Personal interviews, focus group discussions (FGDs), and field observations were employed to gather information on the goat production systems and farmers' perceptions. The mean ± SD of goat flock size in the study area were 7.31 ± 5.89 heads per household (HH) with larger flock sizes observed in the Alaba zone (10.32 ± 6.56). Goats in the studied areas were primarily kept for income generation. The average age at first kidding was 11.3 ± 1.3 months. Relatively better management practices were observed for crossbred goats in the Alaba, Loko Abaya, and Gurage zones. Respondent farmers highly appreciated Boer crossbred goats due to their superior perception of attractive coat color (4.39 times greater, *P* < 0.001), docile behavior (3.59 times greater, *P* < 0.001), fast growth rate (1.64 times greater, *P* < 0.05), and market preference (5.81 times greater, *P* < 0.001). However, susceptibility to disease and drought was considered as drawbacks of crossbred kids in the studied areas. It was also reported that crossbreed goats fetched better prices than indigenous goats of a similar age group and under the same management system. All visited farmers expressed a strong interest in crossbreeding. Based on these findings, it can be concluded that Boer crossbred goats perform well in southern Ethiopia. Therefore, the continued production of crossbred kids can be disseminated to these areas. Additionally, it is suggested to consider the interests of goat producers in the remaining areas. Integrated improved management systems need to be implemented to enhance the survival of crossbred kids. Sustainable training programs should be organized for goat keepers, focusing on aspects such as crossbreeding, minimizing inbreeding, buck rotation, and improved feeding and management practices.

## 1. Introduction

Southern Ethiopia is known for its wide range of indigenous goat breeds. These breeds have adapted to the specific environmental conditions and cultural practices of the region. As documented by CSA, 2021/2022, there was a population of 5,944,175 goats in southern Ethiopia [1]. The goat breeds in this region are generally categorized as part of the broader group of Ethiopian indigenous goat breeds. However, there has been no detailed study on the categorization of these goat breeds. Nevertheless, an old study indicates that the goat population in the Sidama region and Wolaita zones is categorized under Arsi-Bale and Woyto-Guj breeds, respectively [2, 3].

Ethiopia has developed a livestock master plan roadmap with the objective of enhancing the productivity of its livestock sector. Within these plans, there have been ongoing efforts to intensify sheep and goat production, which include crossbreeding with exotic breeds to increase productivity [4]. The genetic improvement strategy mainly focuses on crossbreeding indigenous/local goats with exotic sire breeds practice which was initiated in 1960 [5, 6]. For this purpose, Boer goat multiplication and dissemination sites have been established, Boer goat genotypes have been imported, and research on cross-breeding has been ongoing for decades [7]. The objective of Boer goat crossbreeding is to distribute *F*1 kids to individual farmers, groups of smallholder farmers, and pastoralists [8]. These *F*1 kids are intended to be crossed with indigenous does, resulting in the production and marketing of crossed animals [5, 9, 10].

Jinka Agricultural Research Center (JARC) in southern Ethiopia plays a crucial role in the multiplication and dissemination of crossbred Boer goat bucks and does. Crossing of exotic Boer Goats with the local Woyto-Guji goat breed has been conducted at the Keyafer Breed Evaluation and Distribution (BED) site within JARC [9, 10]. Woyto-Guji goats, known for their productive performance in the region, serve as the foundation for this crossbreeding initiative [11].

The distribution of crossbreed Boer goats was initiated in 2012 and has been ongoing ever since. The research center aims to enhance growth performance and meat production by crossing Woyto-Guji goats with improved Boer goats. To achieve this, the research center imports pure 100 percent Boer breeds from South Africa and conducts on-station crossings with indigenous Woyto-Guji goats. The resulting offspring are *F*1 crossbred 50% Boer bucks and does, which are then multiplied, and disseminated 50% crossbred bucks to farmers for further crossbreeding with indigenous goats [8].

In addition to the crossbred goats, the research center also maintains a population of 100% purebred Boer goats. This is essential for preserving pure breed within the center and supporting the ongoing crossbreeding program. However, it is important to note that these 100% purebred Boer goats are not distributed to farmers. The primary focus is on the dissemination of the crossbred bucks and does, which are expected to contribute to the overall improvement of goat performance and productivity in the region, with a particular emphasis on growth and meat production [8, 12, 13].

However, previous research on goat crossbreeding does not provide sufficient information to offer extension advice for crossbreeding in specific production environments [9, 10, 14]. Therefore, this study aimed to evaluate the impact of 50% x indigenous goat crossbreeding and gather farmer's perceptions about crossbred kids, with the goal of supporting the design of effective future goat crossbreeding programs in southern Ethiopia.

## 2. Materials and Methods

### 2.1. Study Areas

**Figure 1:**
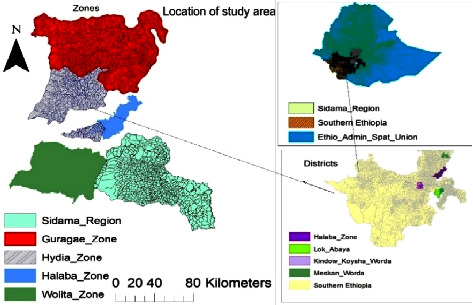
Map of study areas.

Jinka Agricultural Research Center has distributed crossbred Boer goats to South Nation Nationalities and Peoples Regional State (SNNPRS), Sidama Regional State, Oromia Regional State, Dire Dawa town, Hawassa University, and Alemaya University. However, the current study was conducted in five districts of southern Ethiopia and Sidama Regional State. Specifically, Alaba Zuria district (GPS coordinates: 6.0144°N latitude and 37.5509°E longitude), Miskan district (GPS coordinates: 5.9240°N latitude and 36.5200°E longitude), East Badawacho district (GPS coordinates: 6.5320°N latitude and 37.8645°E longitude), and Kindo Koyesha district (GPS coordinates: 6.8359°N latitude and 37.2263°E longitude) were purposively selected from Alaba, Gurage, Hydia, and Wolaita zones, respectively, within the SNNPR. Additionally, Loko Abaya district (GPS coordinates: 6.2676°N latitude and 38.0009°E longitude) was selected from Sidama Regional State (Figure 1). All the study areas were chosen based on the distribution area of Boer crossbreed goats. It is important to note that all study sites are recognized for their goat production potential.

### 2.2. Data Collection Methods and Procedures

A cross-sectional study was conducted from December 22, 2019, to February 13, 2020, to select both study areas and beneficiary farmers. The survey was conducted with Boer cross-goat beneficiary farmers. Study sites, number of distributed crossed animals, date of animal birth, date of animal transfer, and animal receiver were identified from JARC, marking the first stage of data collection. In the second stage, a list of participants in the crossbreeding program was obtained from each selected district office of agriculture and distributor projects (operational research technology dissemination project and Farm Africa). Respondent farmers for interviews were purposively selected from each village. These participants either currently owned Boer crossbreds at the time of the survey or had prior experience with the crossbreeding program. This selection criterion ensured that the participants had direct involvement and relevant knowledge pertaining to the study objectives. In the third stage, semistructured interviews were administered to a total of 100 randomly selected farmers (20 from each district) to assess goat production system, flock structure, purpose, management practices, adaptability, productivity, and their perception of crossbred goats. The traits perceived by farmers were assessed through interviews, and each respondent was asked to assign a preference rank on a scale of 1 to 5 (1 = very poor, 2 = poor, 3 = medium, 4 = good, and 5 = very good) for the productive traits, adaptability, and reproductive measures of both indigenous and crossbred goats. Additionally, farmers were consulted to estimate the market price for both genotypes. The price estimation was based on the aforementioned years.

**Figure 2:**
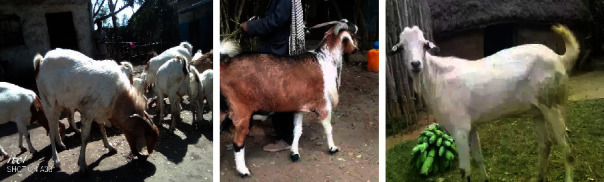
Sample picture of Boer-crossed goat.

To validate and enhance the information gathered from individual interviews, field observations, and FGDs were conducted in each village. Participants for the FGDs were identified based on the preliminary participant list obtained from district's office of agriculture. Key informant interviews were also conducted with each district's Irish Aid Operational Research and Technology Distribution (ORTD) project coordinator and district small ruminant professionals to gather insights on the current status of the crossbreeding program, challenges encountered, and the future prospects of the program. Sample pictures of crossed goats are shown in Figure 2.

### 2.3. Data Analysis

The collected data from respondent farmers were analyzed using the statistical package for social science (SPSS) version 20 [15]. Descriptive statistics such as frequencies, mean, standard deviation, and percentages were used to summarize and present goat keeper's sociodemographic characteristics, flock structure, perception score, breeding practice, and housing system. Chi-square (*χ*^2^) tests were used to assess statistical significance of goat keepers' breeding practice. Additionally, ordinal regression with cumulative logit function was used to examine odds of farmers' perceptions for the two genotypes. Mann–Whitney *U* test was conducted to test for significant differences between the two-genotype reproductive performances. Purpose of keeping goats, source of water, attributes used to determine selling price of goats, and major constraints were ranked and summarized into an index as weighted averages, as suggested by the authors in [16].

Index = sum of (3 for rank 1 + 2 for rank 2 + 1 for rank 3) given for an individual reason (attribute) divided by the sum of ranks (3 for rank 1 + 2 for rank 2 + 1 for rank 3) for overall reasons (attributes). FGDs were open-ended questions and used for discussion purposes.

## 3. Results and Discussion

### 3.1. General Characteristics of Households

The results of demographic and socioeconomic characteristics of selected HHs considered for the survey study are shown in Table 1. The average age of respondent households was 38.34 ± 0.84 years. The mean family size of visited households (±SD) was 7.8 ± 0.43, 6.5 ± 0.4, 7.6 ± 0.43, 7.15 ± 0.4, and 7.8 ± 0.43 for the Alaba, Gurage, Hydia, Sidama, and Wolaita zones, respectively. The average family size of the respondents in the entire study was 7.3 ± 0.18. A large number of family sizes for the survey study were found in the Wolaita and Alaba zones (7.8 ± 0.43), whereas a relatively small number of family sizes for the survey study (6.5 ± 0.43) were found in the Gurage zone. The most frequently distributed sex of the participants in the study was male than female with the proportion of 85 and 15% in that order. The study by the authors in [6] indicates 93% of the interviewed respondents were males which is a little bit higher than the current study. The current result indicates that a relatively better percentage (15%) of women have participated in adopting new technologies in southern Ethiopia. About 45% of the current respondents were able to read and write. However, 35% of respondents were illiterate.

The educational level had significant importance in adopting new technologies and innovations in the communities. In this regard, more work needs to be done by the Ministry of Education on farmer education. Almost 100% of the respondents were involved in the crop-livestock production system. The average land holdings at Alaba, Gurage, Hydia, Sidama, and Wolaita were 1.8, 0.64, 0.28, 1.2, and 1.18 ha, respectively. From the study areas, large land holdings were found in the Alaba Zone. The average landholding was 1.05 ± 0.07 ha. Major attributed to small landholding could be large family numbers, degraded land, and unsuitable land use. A similar study was reported by the authors in [17] in Burundi, where it was observed that larger family sizes tend to allocate more resources towards consumption and developmental activities rather than investing in the modernization of agriculture.

### 3.2. Flock Structures

The overall mean (±standard deviation) of both indigenous and Boer crossbred goats was 7.31 ± 5.89. Comparatively large number of goats (10.32 ± 6.56) was found in the Alaba zone, while a smaller number of goats (3.6 ± 1.67) was recorded in the Hydia zone. The mean (±SD) of crossbred Boer goats was 4.21 ± 4.31. Comparatively large number of crossbreds (7.37 ± 5.7) was recorded in the Alaba zone, while the lowest number of crossbreds (2.10 ± 0.1.21 vs 2.89 ± 1.95) was recorded in Gurgae zone and Hydia zones, respectively (Table 2). Compared with the current study [10], a relatively large number of both indigenous and Boer-crossed goats were kept in northern Ethiopia, Raya Kobo, Habru, and Amhara Sayint districts of Amhara regional state. The difference in flock size of both genotypes might be due to agro ecological differences, size of browsing area, number of Boer buck distribution, follow-up, management, and difference in livestock-keeping experience gathered over a long period transmitted from ancestors [18].

**Figure 3:**
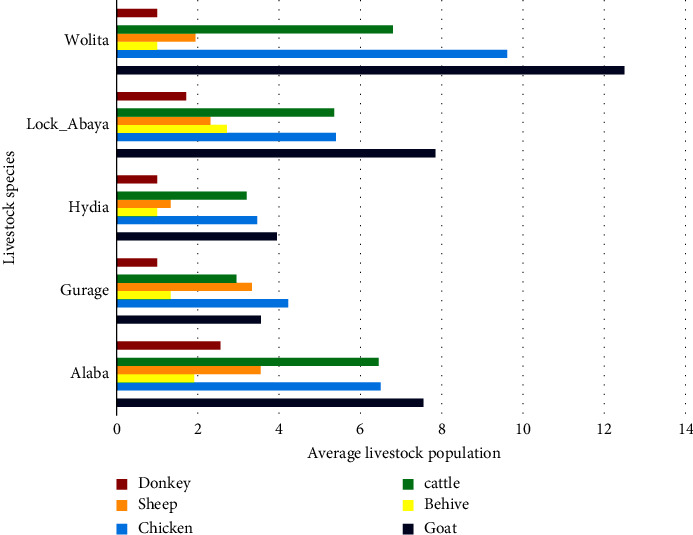
Average number of livestock species per house hold.

Ninety-nine percent of the respondent farmers owned cattle (*n* = 4.97 ± 4.33), 80% owned chickens (*n* = 5.28 ± 4.58), 51% owned donkeys (*n* = 1.66 ± 1.05), and 27% owned beehives (*n* = 1.9 ± 1.11). The indigenous goat population accounted for 53.03% of all goats in the visited flocks. The distribution of livestock species per household is presented in Figure 3. According to the responses from the respondents in all study sites, the goat population was higher in number except from the Gurage and Hydia zones compared to other livestock species.

### 3.3. Purpose of Keeping Goats

Purposes of keeping goats are presented in Table 3. The primary goal for keeping goats was income generation (index = 0.27), followed by savings (index = 0.23). A smaller proportion of respondents kept goats for meat (index = 0.12) and milk (index = 0.12) purposes. A study also revealed that the majority of goat keepers utilized goat milk for their children [19, 20]. Among the respondents, approximately 55% had sold their crossbred goats in the year preceding the survey. The main reasons for selling were to meet household needs (35%), purchase necessities for children (41.6%), and for other purposes (23.4%). A similar purpose of keeping goats and sheep was reported in a previous study by [9, 10]. Generally, the primary purpose of keeping goats in many parts of Ethiopia is income generation, specifically to address emergency cases, cover educational fees, and meet other household expenses [21].

### 3.4. Farmers' Perception on Crossbred Goats

Table 4 demonstrates the relationship between farmers' characteristics, education, age, and their perception of Boer goats. Individuals with education beyond secondary school generally have a positive perception of crossbreed goats, with a mean perception score of 4.5 and low variation in scores. Those with adult education have relatively neutral perception, with a mean score of 2.0 and low variation. The illiterate group had a mean score of 2.0, with a wide range of variations in perception scores. People with primary education have slightly lower positive perception, with a mean score of 3.04 and low variation. Those with reading and writing skills had a more negative perception, with a mean score of 1.9.

The religious group had a consistent perception score of 3.0 but with zero standard deviation due to its small size. Individuals with secondary education have a positive perception similar to those with education beyond secondary school. However, there was no significant difference of the perception score (*P* > 0.05) between respondents' age groups.

These findings suggest that the education level is an important factor that influences farmers' perception of crossbreed results. Farmers, those with higher education levels, tend to have a more positive view of the benefits and outcomes associated with crossbreeding initiatives. The observed relationship between farmers' characteristics and their perception of crossbreed results underscores the importance of targeted extension services and educational programs. Tailoring interventions to address the specific needs and preferences of different farmer groups can enhance the adoption and success of cross-breeding practices. For instance, providing educational resources and training programs targeted at older farmers or those with lower educational attainment may help bridge the perception gap and promote greater acceptance and understanding of the advantages of crossbreeding. It is worth noting that this study focused solely on age and education level as farmers' characteristics. Further research could explore additional factors, such as farming experience, access to information, and socioeconomic status, to gain a more comprehensive understanding of the various influences on farmers' perception of crossbreed goat results [10].

Moreover, understanding community's views, preferences for animals/traits, environments, and trait priorities is essential for the success of indigenous small ruminant genetic improvement [5, 9]. The perceptions of farmers regarding the two genotypes are presented in Table 5. The results indicate that farmers hold significantly different perceptions of crossed and indigenous goats based on coat color, growth rate, goat behavior, twining rate, heat tolerance, drought tolerance, disease tolerance, feed requirement, browsing ability, and market preference. Respondent farmers expressed a greater appreciation for Boer crossbred goats due to their attractive color (4.39 times higher, *P* < 0.001), docile behavior (3.59 times higher, *P* < 0.001), fast growth rate (1.64 times higher, *P* < 0.05), better twining rate (1.73 times higher, *P* < 0.05), and higher reference at market (5.81 times higher, *P* < 0.001). According to the farmers' responses, indigenous goats were perceived to have advantages in terms of heat tolerance, drought tolerance, and disease tolerance. Farmers also reported that crossbred kids have higher feed requirements (2.20 times higher *P* < 0.001) as compared to indigenous ones. In line with the current study, the authors in [10] reported that Boer crossbred goats are more likely to be preferred by farmers than indigenous goats for their growth rate and market value [22]. Regarding the studied locations, the reports from both respondents and discussants across all the sites consistently highlight the perceived superiority of crossbred goats in terms of performance traits and suitability for crossbred kids, including factors such as milk yield, meat quality, and growth rate. However, it is important to note that these findings are based on subjective perceptions rather than measured data. Therefore, there is a need for on-farm empirical data to provide concrete evidence for these claims in future studies.

Furthermore, there are existing on-station and on-farm studies that support the current study's findings. These studies demonstrate better growth, reproductive performance, and survival rates for crossbred goats and their kids [8, 10, 22–25]. The collective evidence from these studies further strengthens the case for the performance advantages of crossbred goats and adds credibility to the results of the current study.

The results of FGDs and key informant interviews indicated that crossbred kids require a more comprehensive management system and regular monitoring compared with indigenous goats. Nearly all participants explained that crossbred kids could outperform indigenous goats if they received proper management, including appropriate feeding, healthcare, and housing sanitation. However, a high incidence of disease was reported in the Hydia zone. According to their responses, crossbred goats were frequently affected by parasites, fungal diseases, and unidentified illnesses, and once affected, it was challenging to rescue the affected animals. Therefore, appropriate attention should be given to crossbred goats to prevent stress caused by parasites, diseases, and inadequate nutrition [5, 9, 10]. Generally, crossbred kids were relatively better accepted by the farmers, while indigenous kids were also preferred due to their resistance to diseases. This might have affected the market price of the crossbred kids [5, 6]. Generally, goat keepers expressed their willingness to crossbreed their indigenous goats with Boer bucks in the future, indicating the need for further efforts from JARC, the district office of agriculture, and other projects. A significant proportion of farmers also acknowledged the importance of incorporating the Boer breed in the indigenous goat genetic improvement approach. Furthermore, the findings from the FGDs and key informant interviews support the responses provided by the farmers.

### 3.5. Reproductive Traits

The reproductive performance results from the survey are summarized in Table 6. The findings indicate that there was no significant difference (*P* > 0.05) between indigenous and crossbreed male goats in terms of age at first service and kidding interval. However, there was a significant difference (*P* < 0.05) between indigenous and crossbreed goats in age at first kidding, female goat age at first service (in months), and average number of kids born.

The mean age at first kidding for crossbred goats (13.9 months) and female age at first service (10.02 months) were shorter compared with indigenous goats. This difference can be attributed to breed variations. Therefore, crossbred Boer goats demonstrate comparatively better performance than indigenous goats, presenting an opportunity for implementing crossbreeding strategies to achieve rapid genetic improvements. Additionally, the average litter size for indigenous goats (1.32 kids/doe) was smaller than the average number of kids of 1.4 litters/doe for Boer crossbreed goats. Relatively shorter KI and a small number of kids were reported by the authors in [22]. The higher twining rate obtained from Boer-crossed goats could be due to the reproductive potential of the breed. In the study areas, kidding occurred at any time of the year. The overall average weaning age of kids was 2.98 ± 0.22 months, with a range of 1 to 4 months. The variation in performance among the breeds could be attributed to the genetic potential of the breed [10].

### 3.6. Breeding Practices

Approximately 75% of the respondent farmers engaged in the practice of exchanging breeding bucks among beneficiary farmers and farmer training centers. The FGDs revealed that farmers employed various forms of buck exchange. In Alaba, 85% of the respondents provided special management for crossbred breeding bucks, while in Loko Abaya, the percentage was 90%. However, in Gurage and Hydia, 80% of the respondents did not provide special management for crossbred animals. In the studied areas, 84% of the respondents reported uncontrolled natural mating as their primary mating practice (Table 7). The main reasons for uncontrolled natural mating were utilization of communal grazing areas where animals from different households of the same flock grazed together, lack of awareness, and insufficient breeding bucks. The majority of the respondents identified their animals based on a combination of naming and color.

### 3.7. Feed Sources

Based on the interviews conducted with the respondent farmers, the main feed resources identified were natural pasture, fallow land, hay, and crop residues. However, during both the wet and dry seasons, natural pasture was the primary source of feed. The availability of feed resources in the studied areas exhibited seasonality. In the Alaba and Gurage zones, crop residues from cereals played a more significant role as feed sources during the dry season when grazing pasture was no longer available. Consequently, seasonal fluctuations in feed supply for goats were more frequently reported in the Alaba and Gurage zones. During the dry season, crop residues such as maize, sorghum, and their stover served as the main feed sources. Additionally, supplementary feeding of crossbred goats was practiced throughout the year, both in the dry and rainy seasons, across all the studied sites. About 40%, 50%, 15%, 35%, and 45% of the farmers in the Alaba, Gurage, Hydia, Loko Abaya, and Wolaita zones, respectively, reported providing supplementary feed during the dry season, particularly from January to May. The lower percentage of supplementary feed in Loko Abaya can be attributed to the presence of large communal grazing lands in the area.

### 3.8. Water Sources and Availability

Table 8 shows the dynamics of water source utilization across different seasons in the study areas. During the dry season, pipe water (index = 0.35) and rivers (index = 0.32) emerge as primary sources, indicating the reliance on more stable water sources when precipitation is limited. This suggests a strategic shift towards dependable water supplies to meet demand during drier periods. Additionally, the low usage of rainwater during this season underscores the challenges of effectively harvesting and storing precipitation. However, the wet season presents a contrasting picture, with a surge in rainwater (index = 0.33) utilization alongside increased reliance on dams/ponds. This shift reflects a harnessing of seasonal abundance, leveraging natural resources such as rainfall and water bodies to supplement water needs. Moreover, the decreased dependence on rivers and pipe water during the wet season suggests a more diversified water portfolio, capitalizing on varied sources to mitigate risks associated with single-point reliance.

The fluctuating patterns observed underscore the importance of adaptive water management strategies tailored to seasonal variations. Planning infrastructure and policies that accommodate the dynamic nature of water availability can enhance resilience to changing conditions. Moreover, these insights can inform sustainable practices for water conservation and equitable distribution, ensuring that communities have consistent access to safe and reliable water throughout the year. By recognizing and leveraging seasonal trends in water source utilization, stakeholders can work towards building more resilient and efficient water systems that address the evolving needs of the study areas.

### 3.9. Housing of Goat

In the studied areas, both family housing and separate housing systems were practiced during the dry and wet seasons. According to Table 9, approximately 49% of the Boer crossbreed user respondents sheltered their crossbred goats inside the family house, while the remaining 51% used a separate house that was located adjacent to the family house. Almost all goats (100%) were housed in structures with wooden walls. In the study zones, goats were housed separately from cattle and equines in 73% of the cases. Additionally, 73% of the kids were housed with adults, while 17% were housed separately in a separate house. Iron sheet roofs are the most common type of roof in all study areas, ranging from 85.0% to 100.0%. Grass roofs are present in Alaba (10.0%) and Loko Abaya (5.0%). Wood roofs are only found in Alaba (5.0%), and concrete roofs are present in Wolaita (5.0%). The majority of houses in all study areas have iron sheet roofs, except for Alaba and Loko Abaya. Mud floors are prevalent in Alaba, Gurage, Hydia, and Loko Abaya, with 100.0%, 0.0%, 0.0%, and 0.0%, respectively. Concrete floors are dominant in Wolaita (95.0%), while a small percentage of houses in Wolaita (5.0%) still have mud floors.

Hydia prefers to house goats within family houses with iron sheet roofs and concrete floors. Wolaita also keeps goats in family houses but has a mix of iron sheets and concrete roofs, with some houses having mud floors. Alaba, Gurage, and Loko Abaya prefer separate goat housing with iron sheet roofs and concrete floors, except for some grass roofs in Alaba and Loko Abaya.

Concrete floors are the most common type, with a majority of houses having those (99.0%). The current result is comparable to [26] findings in Alaba, southern Ethiopia. The authors in [26] reported that 98.6% of respondents confined their sheep and goat flocks in the main house with family members, while 0.7% confined them in separate houses. In the current study, 5.0% of goats in Alaba were housed in family houses, while 95.0% were housed in separate houses. This indicates a higher percentage of goats in separate houses compared to [26] report. However, our result disagrees with that of the authors in [27] report in Ziquala district. The authors in [27] found that the majority (83.82%) of farmers confined their goats without a roof, while a small proportion (18.18%) confined them in the family house. In the current study, 10.0% of goats in Alaba were housed with grass roofs, and 85.0% had iron sheet roofs. This suggests a different housing pattern compared to [27] findings.

### 3.10. Health Management

Respondent farmers in the study area were able to identify the major diseases that affect goat productivity. These diseases include ovine pasteurellosis, external/internal parasites, anthrax, foot and mouth disease, and shoat pox, with index values of 0.31, 0.20, 0.15, 0.15, and 0.19, respectively. In the study areas, the majority of farmers relied on modern drugs obtained from government and private clinics to combat these diseases. The confirmation of this result was supported by the responses obtained from the FGDs and key informant interviews. Tibbo [28] documented similar findings in the North Shewa zone of Ethiopia.

### 3.11. Marketing

According to the responses from the respondents, goats were sold to local traders, consumers, and individual farmers. It was reported that Boer crossbred goats reached a better price than indigenous goats. This indicates that crossbred Boer goats provide greater opportunities for benefiting farmers than indigenous goats.

In the study areas, the accessibility and availability of markets were not reported as issues for Boer crossbred goats. This was confirmed through the FGDs, which further highlighted the existence of a price difference between indigenous goats and Boer crossbred goats. Furthermore, when considering female goats within specific age groups, both crossbred Boer goats and indigenous goats were sold at different prices. Specifically, for goats aged 3–6 months, the prices were 1089 ETB/head versus 770 ETB/head, for goats aged 6–9 months, the prices were 1308 ETB/head versus 1177 ETB/head, for goats aged 9–12 months, the prices were 2253 ETB/head versus 1656 ETB/head, and for goats aged above 12 months, the prices were 2724 ETB/head versus 1904 ETB/head. These results indicate that within the same age group, the price of female crossbred goats consistently exceeded that of local goats by a margin ranging from 131 to 820 ETB/head, considering that both groups were managed similarly.

**Figure 4:**
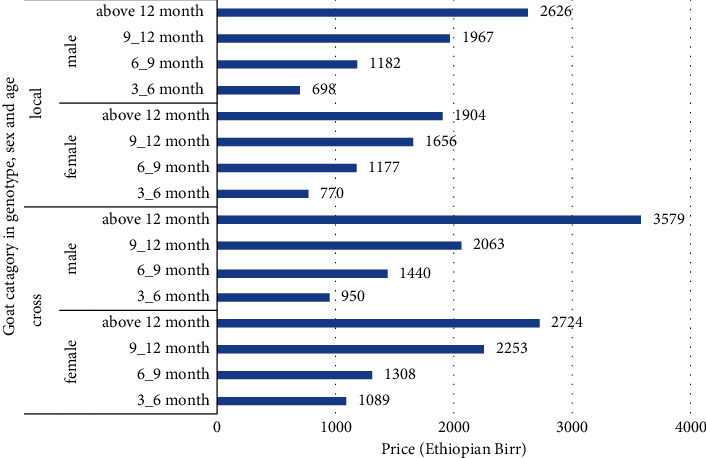
Boer goat price advantage (ETB).

Similarly, for male goats in the corresponding age groups, crossbred Boer goats and indigenous goats were sold for 950 ETB/head versus 698 ETB/head, 1440 ETB/head versus 1182 ETB/head, 2063 ETB/head versus 1967 ETB/head, and 3579 ETB/head versus 2626 ETB/head, respectively, as shown in Figure 4. The price difference per genotype for male goats ranged from 96 to 953 ETB/head. Within a similar age group, male crossbred Boer goats aged above 12 months exhibited a higher price difference of 722 ETB/head compared with female local goats. However, it is important to note that the respondents provided estimates for the market prices of both genotypes, and these estimates were based on the year 2019/2020. These findings align with a study by the authors in [10] which reported that the price of Boer × Central Highland crossbred goats was higher (by 213 to 372 ETB/head) than Central Highland goats with similar management and age.

### 3.12. Attribute Used for Determining Selling Price

The main attributes used to determine the selling price of goats were physical appearance, coat color, animal age, and animal sex. These attributes were ranked in order of importance, with physical appearance being ranked first, followed by coat color, animal age, and animal sex, with index values of 0.36, 0.30, 0.17, and 0.16, respectively (see Table 10). Similar findings were reported by the authors in [5, 6] in the North Wollo Zone of Amhara region, Ethiopia.

### 3.13. Main Constraints of Crossbreeding

The crossbreeding constraints, as identified by goat keepers in the study areas, have been presented in Table 11. The most significant constraints for crossbreeding program and crossbred goats were disease susceptibility (0.16), lack of genotype (0.15), lack of capital (0.14), and poor adaptability (0.14), ranking as the first, second, third, and fourth, respectively. These findings are supported by the authors in [8], who reported that approximately 66.7% of their respondents stated that the lack of disease resistance in Boer crossbred kids was more prevalent compared to their local kids in Boer x Woyito-Guji crossbreed goats in the Hammer district of agropastoral South Omo Zone, Ethiopia. However, in contrast to the current results [10], it was reported that feed shortage was the primary challenge mentioned by goat keepers in the northeastern part of Ethiopia regarding Boer goat crossbreeding. The author also noted that 77.8% of the respondents did not prefer crossbreeding due to the poor adaptability of the crossbred kids, specifically in Habru Woreda.

## 4. Conclusions

Goats are the predominant animal species kept in the study areas and play a crucial role in the livelihoods of farmers. Boer crossbred goats have gained popularity among farmers due to their attractive coat color, desirable behavior, rapid growth rate, twinning rate, and market demand, surpassing those of indigenous goats. Farmers who utilize crossbred bucks or cross their does with Boer crosses can obtain exceptional offspring, thereby commanding higher prices in the market. These offspring are characterized by appealing coat color, enhanced growth performance, and sound phenotypic characteristics. However, these farmers have reported encountering challenges such as the susceptibility of bucks obtained to diseases and difficulties in acquiring crossbred bucks from the research center. This is primarily due to the significant number of people in the queue. It is crucial to focus on improving veterinary services and enhancing the multiplication and availability of Boer crossbred bucks. In the study area, Boer crossbred goats have limitations, including their relative vulnerability to diseases and limited tolerance to drought and heat. Nevertheless, farmers still prefer crossbred goats due to their overall adaptability and productivity. As a result, farmers have expressed the need for Boer goats to be incorporated into crossbreeding programs with indigenous goats to address these concerns.

## Figures and Tables

**Table 1:** General information of respondents.

Respondents	Alaba	Gurage	Hydia	Sidama	Wolaita	Overall
Age of respondents (*N* = 100)	37.5 ± 1.8	35 ± 1.8	37.9 ± 1.8	38.4 ± 1.8	42.8 ± 1.8	38.34 ± 0.84

Family size (*N* = 100)	7.8 ± 0.43	6.5 ± 0.4	7.6 ± 0.43	7.15 ± 0.4	7.8 ± 0.43	7.3 ± 0.18

Landholding (ha)	1.8	0.64 ± 0.1	0.28 ± 0.2	1.2 ± 0.1	1.18 ± 0.1	1.05 ± 0.07

Sex *N* = 100	Female	10.0%	30.0%	25.0%	15.0%	15.0%
	Male	90.0%	70.0%	75.0%	85.0%	85.0%

Respondents' educational level *N* = 100	Above secondary	20.0%	0.0%	0.0%	15.0%	0.0%
	Adult education	0.0%	5.0%	0.0%	5.0%	0.0%
	Illiterate	25.0%	15.0%	30.0%	30.0%	35.0%
	Primary (1–8)	25.0%	25.0%	45.0%	35.0%	15.0%
	Reading and writing	10.0%	5.0%	20.0%	5.0%	45.0%
	Religious	5.0%	5.0%	0.0%	0.0%	0.0%
	Secondary	15.0%	45.0%	5.0%	10.0%	5.0%

**Table 2:** Flock size and structures in the study areas.

Study area	*N*	Flock size						
		Indigenous			Crossed			Average (mean ± SD)
		Min	Max	Mean ± SD	Min	Max	Mean ± SD	
Alaba	20	1	10	3.80 ± 3.05	1	24	7.37 ± 5.70	10.32 ± 6.56
Gurage	20	1	7	2.74 ± 2.25	1	6	2.10 ± 0.21	4.70 ± 2.69
Hydia	20	1	3	2.19 ± 0.75	1	8	2.89 ± 1.95	4.58 ± 2.69
Loko Abaya	20	4	21	8.55 ± 4.18	2	8	4.63 ± 1.77	8.53 ± 3.42
Wolaita	20	1	16	5.65 ± 0.64	1	27	4.07 ± 6.70	8.50 ± 9.09
Average flock size	100	1	21	3.44 ± 0.32	1	27	4.21 ± 4.31	7.31 ± 5.89

**Table 3:** Purpose of keeping a goat in the study area.

Purpose of keeping goats	Ranking index											
	Alaba		Gurage		Hydia		Loko Abaya		Wolaita		Over all	
	Index	Rank	Index	Rank	Index	Rank	Index	Rank	Index	Rank	Index	Rank
Income	0.27	1	0.25	1	0.26	1	0.27	1	0.31	1	0.27	1
Meat	0.19	2	0.04	4	0.26	1	0.11	4	0.03	3	0.12	3
Milk	0.08	3	0.15	2	0.23	2	0.11	4	0.02	4	0.12	3
Manure	0.07	4	0.08	3	0.02	5	0.16	3	0.29	2	0.12	3
Skin	0.01	5	0.01	5	0.05	4	0.01	5	0.03	3	0.02	4
Saving	0.19	2	0.25	1	0.15	3	0.24	2	0.31	1	0.23	2

**Table 4:** Farmers' characteristics and perceptions of Boer goat crossbreeds by educational level and age group.

Respondents	*N*	Perception score (mean ± SD)
Educational level	*P* < 0.001	
Above secondary school	7	4.5 ± 0.53
Adult education	3	2.0 ± 0.20
Illiterate	22	2.0 ± 1.02
Primary (1–8)	23	3.04 ± 0.56
Reading and writing	25	1.9 ± 0.52
Religious	2	3.0 ± 0.00
Secondary	19	4.00 ± 0.66
Total	100	2.79 ± 1.16
Age group (years)	*P* > 0.05	
31_40	45	2.84 ± 1.27
41_50	24	2.68 ± 1.14
51_60	3	2.0 ± 0.00
Greater than 65	3	2.50 ± 0.70
Less than 30	25	2.92 ± 1.03
Total	100	2.80 ± 1.16

Note: perception scores were measured on a scale of 1 to 5, with 1 indicating “strongly disagree” and 5 indicating “strongly agree.” Higher scores indicate a more positive perception of crossbreed results.

**Table 5:** Traits perceived by farmers for local and crossbred goats.

Variables	Breed	Very poor (%)	Poor (%)	Medium (%)	Good (%)	Very good (%)	Cumulative odd ratio
Physical appearance	Local	4.16	6.38	32.97	35.4	21.87	1.00
	Cross	10.46	3.48	5.81	51.16	29.06	0.85^ns^

Color	Local	0.0	2.12	2.12	71.27	24.46	1.00
	Cross	20.21	5.31	14.89	37.23	22.34	4.39^*∗∗∗*^

Behavior	Local	11.11	10.0	10.0	51.11	17.77	1.00
	Cross	20.0	22.22	7.77	16.66	33.33	3.59^*∗∗∗*^

Growth rate	Local	12.0	8.0	46.0	21.0	13.0	1.00
	Cross	20.45	0.0	6.8	28.4	44.31	1.64^*∗∗*^

Meat quality	Local	19.76	11.62	10.46	50.0	8.13	1.00
	Cross	3.26	5.43	15.21	54.34	21.73	1.12^ns^

Twining rate	Local	1.09	5.49	42.85	25.27	25.27	1.00
	Cross	28.57	6.59	15.38	36.26	13.18	1.73^*∗∗*^

Milk yield	Local	17.07	32.92	32.92	12.30	4.87	1.00
	Cross	1.10	1.10	36.2	27.7	34.0	1.10^ns^

Heat tolerance	Local	6.81	3.40	1.13	25.0	63.63	1.00
	Cross	14.77	31.81	13.63	34.09	5.68	0.44^*∗∗*^

Drought tolerance	Local	16.66	1.04	4.17	25.00	53.12	1.00
	Cross	24.75	26.73	23.76	9.90	14.85	0.43^*∗∗*^

Disease tolerance	Local	0.00	0.00	0.00	7.00	93.00	1.00
	Cross	34.04	35.10	25.53	1.06	4.25	0.48^*∗∗*^

Feed requirement	Local	23.6	6.7	16.9	52.8	0.0	1.00
	Cross	35.7	0	4.8	23.8	35.7	2.20^*∗∗*^

Browsing ability	Local	2.17	1.08	6.52	3.26	86.95	1.00
	Cross	14.28	4.39	15.38	40.65	25.27	0.28^*∗∗∗*^

Preference at market	Local	0.0	14.60	33.70	20.22	31.46	1.00
	Cross	17.04	1.13	11.36	7.95	62.5	5.81^*∗∗∗*^

1.00, reference, ^*∗∗∗*^ = *P* < 0.0001, ^*∗∗*^ = *P* < 0.05, ns ≥ 0.05.

**Table 6:** Reproductive performances of cross and indigenous goats.

Parameters	*N*	Crossed			Indigenous			Difference	P (Mann Whitney *U* test)
		Min	Max	Mean ± SD	Min	Max	Mean ± SD		
AFK (months)	186	12	20	13.90 ± 4.60	12	24	16.49 ± 3.00	−2.52	0.04
Male AFS (months)	185	6	23	10.70 ± 2.00	7	22	10.59 ± 3.01	0.11	NS
Female AFS (months)	162	7	15	10.02 ± 5.50	9	30	10.56 ± 4.43	−0.54	0.02
ALS (kids)	176	1	3	1.40 ± 0.40	1	3	1.32 ± 0.07	0.07	0.04
ALS/lifetime	95	—	—	—	4	24	12.34 ± 5.03	—	Unable to compute
KI (months)	180	7	11	6.83 ± 2.40	7	12	7.30 ± 2.70	−0.53	NS

AFK = age at first kidding, AFS = age at service, ALS = average litter size, KI = kidding intervals, NS = nonsignificant.

**Table 7:** Farmer's response to goat breeding management.

Questions		Zones					Total	*P* value
		Alaba (%)	Gurage (%)	Hydia (%)	Loko_Abaya (%)	Wolaita (%)		
Do you practice cross breeding	No	10.0	0	30	10.0	5.0	15.0	<0.001
	Yes	90.0	100.0	70.0	90.0	95.0	85.0	

Sources of breeding bucks	FTC	40.0	0	5	5.0	0	9.0	<0.001
	Inherited	10.0	0	0	10.0	0	4.0	
	Marketed	5.0	0	5.0	0	0	2.0	
	Neighbors	10.0	0	10	15.0	10.0	7.0	
	NGO	35.0	100.0	80	70.0	90.0	74.0	

Is there a buck exchange?	No	10.0	20.0	70.0	25.0	0	25.0	<0.001
	Yes	90.0	80.0	30.0	75.0	100.0	75.0	

Is there any special management for breeding bucks?	No	15.0	80.0	80.0	10.0	45.0	41.0	<0.001
	Yes	85.0	20.0	20.0	90.0	55.0	59.0	

Mating type	Controlled	0	10.0	0	25.0	0	8	<0.001
	Uncontrolled (hand mating)	20.0	0	5	20.0	0	8.0	
	Uncontrolled (natural mating)	80.0	90.0	95.0	55.0	100.0	84.0	

Goat identification	Coat color	20.0	5.0	10.0	0	0	0	<0.001
	Combination of all	80.0	70.0	70.0	40.0	85.0	70.0	
	Naming	0	25.0	20.0	60.0	15.0	30.0	

FTC = farmer training center.

**Table 8:** Source of water in the study areas in different seasons.

Sources	Dry season			Index	Wet season			Index
	First	Second	Third		First	Second	Third	
Water well	7	1	0	0.04	2	0	3	0.03
Dam/pond	7	6	6	0.09	1	1	13	0.08
River	26	22	17	0.32	8	22	4	0.18
Spring	8	24	4	0.18	2	5	19	0.13
Pipe water	39	24	8	0.35	34	9	7	0.26
Rainwater	2	0	1	0.02	28	31	5	0.33

**Table 9:** Reported housing of goats in the study areas.

Study areas	Housing type		House roof			Floor	
	In family house (%)	Separate house (%)	Grass (%)	Iron sheet (%)	Wood (%)	Concrete (%)	Mud (%)
Alaba	5.0	95.0	10.0	85.0	5.0	0	100
Gurage	10.0	90.0	0.0	95.0	0.0	0	100
Hydia	90.0	10.0	0.0	100.0	0.0	0	100
Loko Abaya	45.0	55.0	5.0	95.0	0.0	0	100
Wolaita	95.0	5.0	0.0	100.0	0.0	5.0	95
Total	49.0	51.0	3	95	1	1.0	99

**Table 10:** The main attribute used for determining the selling price of goats.

Criteria	Ranking					Index	Rank
	R1	R2	R3	R4	R5		
Physical appearances	365	48	6	0	0	0.36	1
Coat color	30	260	48	10	0	0.30	2
Age	0	16	108	70	2	0.17	3
Sex	5	16	60	90	14	0.16	4

**Table 11:** Constraints of Boer goat crossbreeding.

Constraints	Index	Rank
Disease's susceptibility	0.16	1
Lack of genotype	0.15	2
Lack of capital	0.14	3
Poor adaptability	0.14	3
Feed shortage	0.13	4
Drought	0.11	5
Water shortage	0.08	6
Market preference	0.04	7
Labor shortage	0.04	7

## Data Availability

Data will be made available on request from the corresponding author.
